# Characterization of Human Colorectal Cancer MDR1/P-gp Fab Antibody

**DOI:** 10.1155/2013/716289

**Published:** 2013-11-07

**Authors:** Xuemei Zhang, Gary Guishan Xiao, Ying Gao

**Affiliations:** ^1^The Medical College of Dalian University, Dalian Economic & Technical Development Zone, Dalian 116622, China; ^2^Department of Biochemistry and Molecular Biology, Dalian Medical University, 9 Western Section, Lvshun South Street, Lvshunkou District, Dalian 116044, China; ^3^Departments of Medicine and Medical Microbiology, Creighton University, 601 N 30th Street, Omaha, NE 68131, USA

## Abstract

In this study, the peptide sized 21 kDa covering P-gp transmembrane region was first prepared for generating a novel mouse monoclonal antibody Fab fragment with biological activity against multiple drug resistance protein P-gp_21_ by phage display technology. Phage-displayed antibody library prepared from mice spleen tissues was selected against the recombinant protein P-gp_21_ with five rounds of panning. A number of clones expressing Fab bound to P-gp_21_, showing neutralized activity in vitro, were isolated and screened by enzyme-linked immunosorbent assay based on its recognition properties to P-gp_21_ and human colorectal cancer tissue homogenate, resulting in identification of an optimal recombinant Fab clone (Number 29). Further characterization by recloning number 29 into an expression vector showed significant induction of the Fab antibody in the clone number 29 by Isopropyl **β**-D-1-thiogalactopyranoside (IPTG). After purified by HiTrap Protein L, the specificity of the Fab antibody to P-gp_21_ was also confirmed. Not only was the targeted region of this monoclonal Fab antibody identified as a 16-peptide epitope (ALKDKKELEGSGKIAT) comprising residues 883–898 within the transmembrane (TM) domain of human P-gp, but also the binding ability with it was verified. The clinical implication of our results for development of personalized therapy of colorectal cancer will be further studied.

## 1. Introduction

Failure of chemotherapy resulting from a multidrug resistance (MDR) remains the major obstacle during cancer treatment. Development of effective screening tools for personalized therapy of cancer will enhance the response rate of the patients to the designated therapeutic regimen. One of the gene products involved in MDR is P-glycoprotein (P-gp). P-gp is a 170 kD plasma membrane efflux pump protein and belongs to the ATP-binding cassette superfamily of transport proteins [1]. Comparative sequence analysis of P-gp with other ABC family members shows that it consists of two transmembrane (TM) domains, each containing six putative TM segments followed by an ATP-binding consensus motif and two cytoplasmic nucleotide-binding domains (NBDs). The TMs of P-gp are found mainly responsible for binding to and transporting a broad spectrum of drugs. Photolabeling and mutational studies indicate that the drug binding domain is within the TM domains of P-gp, and drug transport is powered by hydrolysis of ATP at the two cytoplasmic NBDs [1, 2]. Structure-function analyses of P-gp suggested that two subunits of P-glycoprotein are involved in the selection of its drug substrate and/or specificity of MDR modulator. Crucial amino acid residues of P-gp are localized within or near the TM region [3, 4]. P-gp functions as an efflux pump expelling drugs out of tumor cells, resulting in a decrement intracellular concentration of cytotoxic drugs. Overexpression of P-gp is associated with poor clinical outcome [5–7]. Identification of the tumor cells sensitive to antineoplastic drugs may help for the selection of the antineoplastic agents [8]. Although methyl thiazolyl tetrazolium (MTT) can provide direct observation on the tumor cells sensitive to antitumor drugs, it is a time-consuming process. Methods used extensively for detection of the P-gp protein expression, such as ELISA, immunohistochemical method (IHCA), and flow cytometry (FCM), depend on the reaction of the P-gp protein to its corresponding monoclonal antibody. Nevertheless, use of the intact monoclonal antibodies may provide false positive signals in detection of the tumor cells since the Fc fragments of IgG may bind to the Fc receptors on the surface of normal cells as well. Thus, use of the Fab antibody may be superior to reducing the false signal resulting from the intact antibody [9, 10]. Meanwhile, Fab is smaller than IgG in size, facilitating trafficking of the antigen across cell membrane and may provide more information than the intact antibody during the ICHA process [11, 12].

In this communication, we constructed and characterized the phage-displayed mouse Fab against human MDR1/P-gp.

## 2. Materials and Methods

### 2.1. Animals

The study was conducted in accordance with national guidelines for care and use of animals. All mice were purchased by the Dalian Medical University Laboratory Animal Center (approval number LA 2009-008). The animals used in the study were age-matched, mature BALB/c female mouse weighing approximately 18–22 g.

### 2.2. Construction of Phage-Displayed Mouse Fab Antibody Library against Human Colorectal Cancer MDR1/P-gp

Six BALB∖C female mice were injected subcutaneously (0.2 mL/10 g) with 0.5 mg/mL human colorectal cancer homogenate emulsified in complete Freund's adjuvant (CFA). The same colorectal cancer homogenate and aliquot of Freund's incomplete adjuvant were subcutaneously injected on the seventh and fourteenth days, respectively. Seven days after the last immune, blood samples were collected and used for determination of the antibody titer by ELISA. When the antibody titer reached above 1 : 512, the spleens of the mice were removed, immediately frozen by liquid nitrogen, and used for extraction of total RNA. Specific cDNA was synthesized from RNA samples with random primer using the RevertAid First Strand cDNA Synthesis Kit (Fermentas) according to the manufacturer's instructions. The Fd chain, Lambda chain, and Kappa chain were then synthesized from the cDNA with 37 pairs of primers which were designed to anneal with mouse FR1 and CH_1_ or CL regions with high fidelity Taq DNA polymerase (Fermentas, USA), respectively. PCR amplification was performed for 35 cycles (1 min at 94°C, 1 min at 55°C, and 1 min at 72°C) in a Thermal Cycler S100 (Bio-Rad, CA, USA). 

PCR products were pooled into independent collections of Fd chain, *κ* chain, and *λ* light chain and purified by using an AxyPre DNA Gel Extraction Kit (Axygen, CA, USA). The recombinants with Fd were performed according to the method described as “Antibody Phage Display Methods and Protocols” [13]. The recombinants with light chain and pGEM-T were firstly digested by *Xba I* and *Sac I* (Fermentas, USA), then ligated into the Fd-pComb3 library, and transfected into *E. coli* XL1-Blue cells (Stratagene, CA, USA) repeatedly to obtain the Fab library (>10^6^ CFU). The presence and size of the inserts were further confirmed sequentially with *Xho I/Spe I* and *Xba I/Sac I*.

### 2.3. Cloning and Expression of the P-gp Transmembrane Region

A gene encoding 185 amino acids covering P-gp transmembrane region was isolated from the colon cancer tissues and amplified by PCR using the sense primer 5′  CCGGAATTCCTCACCAAGCGGCTCCGAT 3′ and the antisense primer 5′  CCGCTCGAGGAGTTTATGTGCCACCAAGTAG 3′ and then cloned into a pET28a (+) vector. The recombinant was further verified by sequencing. The positive recombinants were selected and amplified by adding 1.0 mM IPTG for 6 h at 30°C. Cell pellets were collected by centrifugation at 14,006 ×g for 3 min. Protein was extracted from the cell pellets by using the nickel column (GE Healthcare, CA, USA). The purified P-gp_21_ was verified by Western blot with the His-tag monoclonal antibody (1 : 2000) (Protein Tech, CA, USA).

### 2.4. Phage Display and Screening of the Clones

Phagemid rescuing and the Fab displayed library screening were conducted according to the protocol described [14]. Phage particles exposing antibody fragments were rescued from the Fab library with helper phage with VCSM13 helper phage (2.6 × 10^11^ pfu/mL) on a 20 mL scale. The resulting phage mixtures were used for binders selection on Nunc-Immuno MaxiSorp flat-bottomed microtiter plates (Nunc, Denmark) coated with 49 *μ*g/100 *μ*L of P-gp_21_ dissolved in bicarbonate buffer (pH 9.6) per well overnight at 4°C. Coated plates were incubated in 3% BSA in PBS 300 *μ*L per well for 2 hr at 37°C for blocking. Then 200 *μ*L Fab phage particles prepared above were incubated in microtiter plates for 1 h at RT. Nonbound phages were washed away for 1, 5, 10, 15, to 20 times per round with PBS containing 0.1% Tween 20. The bound phage particles were then eluted with 200 *μ*L of elution buffer (0.1 M HCl-glycine, pH 2.2) per well and immediately neutralized with 14 *μ*L of 2 M Tris base solution. Collected phages were used to infect *E. coli* XL1-Blue cells in an exponential state and amplified as described above. Rescued phage particles were used to start a new selection round in the same conditions according to the same protocol as described above [14]. The phagemid titer was checked by counting the colony-forming unit (CFU) of the phagemid infected by XL1-Blue cells just before and after each panning. After the fifth round of screening, a total of 10 colonies were picked randomly and verified by a double digestion using enzymes *Xba I/Sac I* and *Xho I/Spe I*, respectively.

### 2.5. Further Verification of the Anti-P-gp Positive Clones by ELISA

The positive Fab colonies were picked to test their P-gp_21_ recognition properties. Afterwards, aliquots (20 *μ*L) of the positive clones were transferred to 10 mL of LB-Amp-Tet and further cultured until their A600 reached the same as 0.4. Soon after, IPTG was added to a final concentration of 0.05 mM and kept shaking at 220 rpm at 28°C for six hours. The supernatants of each clone after centrifugation at 14,006 ×g for 2 min at 4°C pellets were collected and sonicated for 10 min for ELISA.

Moiety of the purified P-gp_21_ (1 *μ*g/*μ*L) and human colorectal cancer homogenate (2 *μ*g/*μ*L) was incubated in 50 *μ*L bicarbonate buffer (pH 9.6) at 4°C overnight, respectively. After blocking with 3% BSA for 2 hrs at 37°C, an equal of the soluble protein from each clone prepared above was mixed well for 1 hr at 37°C. After washing with 0.1% Tween 20 in PBS, alkaline-phosphatase- (ALP-) labeled horse anti-mouse IgG antibody (1 : 2000) (Vector Laboratories, Inc., CA, USA) was added as the secondary antibody and p-nitrophenyl phosphate (PNPP) as a chromogenic agent was coincubated with each blot. The clone with pComb3 was served as a negative control; meanwhile, the *E. coli* XL1-Blue was served as a black control. The absorbance was measured at 405 nm with a microplate reader (Model 550, Bio-Rad, CA, USA). Meanwhile, the optimal clone was further verified by the analysis of Western blot. Mouse anti-His antibody (1 : 2000) was used as a positive control, and crude cell extract of pComb3 clone was served as a negative control. Horse anti-mouse antibodies conjugated to alkaline phosphatase as the secondary antibody (1 : 2000) and BCIP/NBT (Amresco, CA, USA) as a chromogenic agent. Data obtained from the Western blot were analyzed by Bio-Rad Quantity One 1D Analysis software version 1.1 (Bio-Rad, CA, USA). Plasmid DNA from the optimal clone was purified and the inserts were completely sequenced and the deduced amino acid sequences were compared with DNA databank data using the BLAST program (National Center for Biotechnology Information, USA) to ascertain its sources.

### 2.6. Production of Soluble Fab Fragments

The recombinant plasmid DNA from the clone number 29 was digested with *Spe I* and *Nhe I* (MBI Fermentas, USA) for 2 h at 37°C to remove the gIII fragment from pComb3, purified by using gel electrophoresis, and then self-ligated to build constructs for expression of soluble recombinant Fab. After the recombinant was identified by *Not I* digestion, the clone was suspended in LB medium containing 100 *μ*g/mL of ampicillin and incubated overnight at 30°C. Afterward, 4 mL of this culture was inoculated in 400 mL of LB medium with ampicillin at 100 *μ*g/mL and induced by isopropylthio-b-galactoside at the final concentration of 0.5 mM until the culture reached exponential growth and then further incubated for 8 h at 28°C. The *E. coli* cells were harvested by centrifugation at 2218 ×g for 15 min at 4°C, and the pellet was suspended with 20 mL of PBS and sonicated on ice. Crude cell extract with Fab fragments was obtained by centrifugation at 8,873 ×g for 30 min at 4°C.

### 2.7. Purification of Fab

The supernatant containing Fab prepared above was filtered by 0.22 mm filter membrane. The filtered solution was loaded onto Capto-L agarose chromatography column (HiTrap Protein L, GE) with the flow velocity of 1 mL/min. After washing out the unbound protein, the Fab was eluted out with sodium acetate buffer (pH 2.3) and neutralized with Tris-HCl (pH 8.0) immediately. Both flow-through unbound protein and eluted protein were collected for further verification by SDS-PAGE.

### 2.8. Analysis of the Characters of the Anti-P-gp Fab Fragment Expressed in *E. coli* XL1-Blue

After purification, Fab concentration was determined using a BCA protein assay kit (Pierce Biotechnology, USA). The specificity of the purified Fab to P-gp_21_ was also analyzed by Western blot using goat anti-mouse IgG conjugated to HRP (1 : 3000) (Southern Biotech, CA, USA). The moiety of BSA and the 15 kDa peptide expressed by BL_21_ (prepared by our lab) were served as the negative control. 

**Figure 1:**
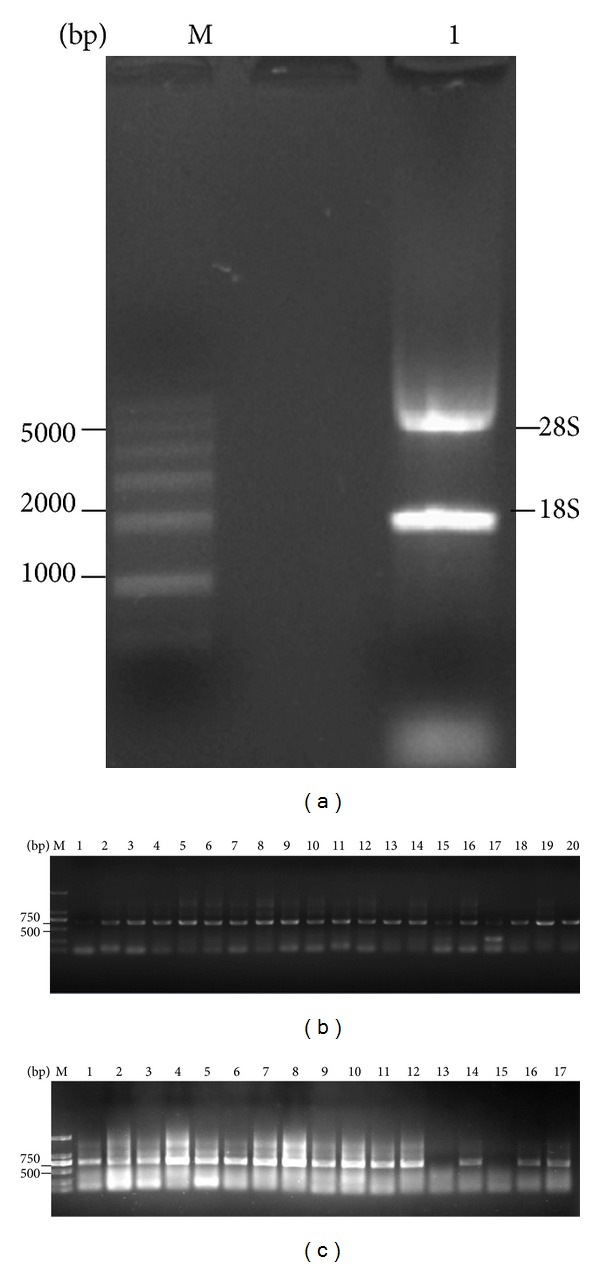
Agarose gel electrophoresis of total RNA and the PCR products of IgG light chains. (a) M: RL6000 RNA marker; lane 1: total RNA of mouse spleen; (b) PCR products of the *κ* chains (lanes 1~18), PCR products of *λ* chains (lanes 19~20); (c) PCR products of the Fd chains (lanes 1~17).

P-gp_21_ harboring three epitopes was chosen as the antigen according to its antigenicity estimated by using BepiPred 1.0b Server (Figure 1). Three peptides with strong antigenicity coupled to bovine serum albumin were synthesized (China Peptides Co. Ltd, Shanghai, China) for selection of the Fab. A 96-well microtiter plate (Nunc, Denmark) was coated with aliquot of BSA, 10-peptide-BSA, 12-peptide-BSA, and 16-peptide-BSA, at 1 *μ*g/*μ*L, and then incubated at 4°C overnight. After washing away unbound antigen, the plate was incubated in the blocking solution (3% BSA in PBS) at 37°C for 1 h. The plate was then incubated with the Fab antibody prepared above (approximately 6 *μ*g/mL) at 37°C for 1 h. After ten-time wash with TBS, the plate was then incubated with fluorescein-isothiocyanate- (FITC-) conjugated goat anti-mouse IgG (Shanghai Meilian Biological Technology Co., Ltd., China) (1 : 1000) as secondary antibody for 1 hour at 37°C. Crude cell extract of pComb3 cell was used as a negative control and the secondary antibodies were used only as a background control in the detection system. The relative fluorescence unit (RFU) was measured by a Multimode Reader (LB941, Berthold Technologies, Germany) with excitation spectrum at 490 nm and emission spectrum at 520 nm.

The specificity of the Fab antibody was measured by a sequential dilution of the Fab fragments immobilized with 16-peptide-BSA and BSA microplates as mentioned above.

## 3. Results

### 3.1. Quantitative Analysis of Total RNA

Total RNA extracted from an immunized mouse spleen was measured quantitatively. The quality of the RNA samples was estimated by using the ratio of A260/A280 and agarose gel electrophoresis. The ratio of A260/A280 for all RNA samples was in the range of 1.8–2.2, suggesting that a highly qualified RNA sample was obtained. Quality of total RNA samples was further verified by 1% agarose gel electrophoresis, showing clear bands in 28S RNA and 18 S RNA (Figure 1(a)).

### 3.2. Construction of Fd Library

To construct IgG heavy chain recombinant Fd-pComb3, we first amplified IgG light chains and Fds by PCR. Figures 1(b) and 1(c) showed that IgG light chains and Fds were successfully amplified with an approximate size of 650 bp and 730 bp separately as shown in 1% agarose gel. Then light chains and Fd fragments were purified for the next library construction. Fd-pComb3 recombinant was constructed, followed by purification and ligation. The library of Fd-pComb3s was transformed into Top 10 competent bacteria by heat shock.

### 3.3. Construction of Fab Library

**Figure 2:**
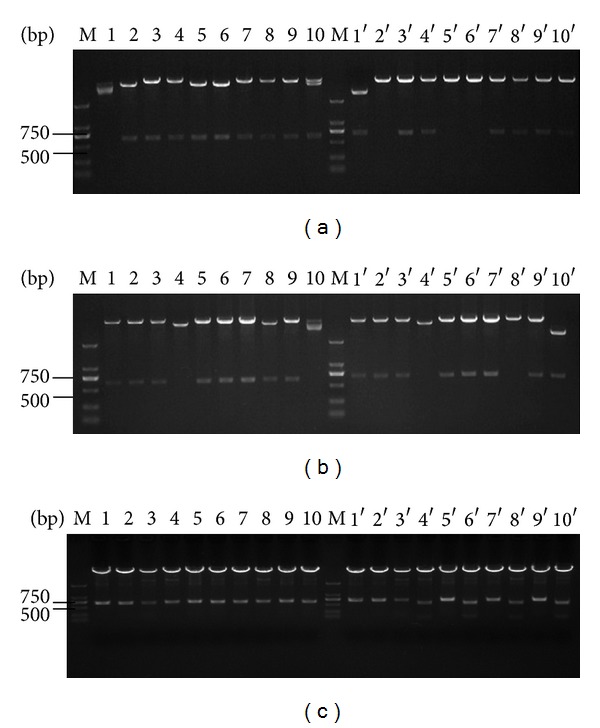
Verification of the Fab clones before and after panning by double-enzyme digestion. (a) Before panning, Fab clones (number 1~number 10) were digested by *Xba I/Sac I *(lanes 1~10) and by *Xho I/Spe I *(lanes 1′~10′); (b) before panning, Fab clones (number 11~number 20) were digested by *Xba I/Sac I *(lanes 1~10) and by *Xho I/Spe I *(lanes 1′~10′); (c) after panning, Fab clones (number 1~number 10) were digested by *Xba I/Sac I *(lanes 1~10) and *Xho I/Spe I *(lanes 1′~10′).

The recombinants were first constructed by connection of the light chains with T carriers and then transformed into TOP 10 cells and cultured at 37°C overnight. During the blue-white selection, when the ratio of white clones/blue clones exceeded 95%, they were collected and reached 1.8 × 10^5^ CFU after several transductions. After digestion of Fd-pComb3 and light chain-T recombinants with *Xba I/Sac I*, the recombinants of Fab-pComb3 were transformed into *E. coli* XL1-Blue successfully, and the final content of the Fab library (>10^6^ cfu) was achieved. Twenty clones were picked from the Fab library randomly and digested by *Xba I/Sac I *and *Xho I/Spe I *simultaneously to verify the insert ratio of Fab (Figures 2(a) and 2(b)). The content of mouse Fab antibody library against human colorectal cancer P-gp was reached by 2.47 × 10^6^ cfu. The insert ratio of the light chain, the Fd chain, and the Fab chain was reached by 90%, 80%, and 72%, respectively.

### 3.4. Cloning of Human Multidrug Resistant Protein P-gp_**21**_

**Figure 3:**
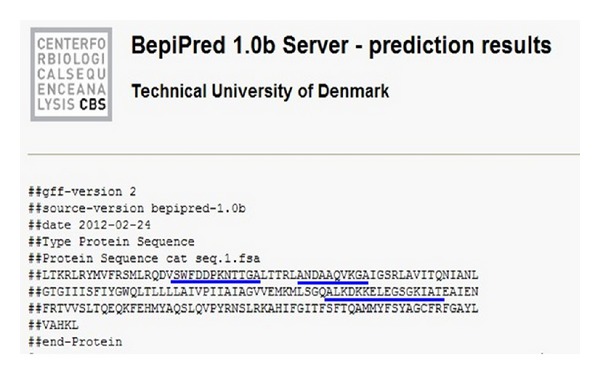
The epitopes analysis of 784~968 amino acids of P-gp. Blue underlines amino acids: the possible epitope of target protein.

**Figure 4:**
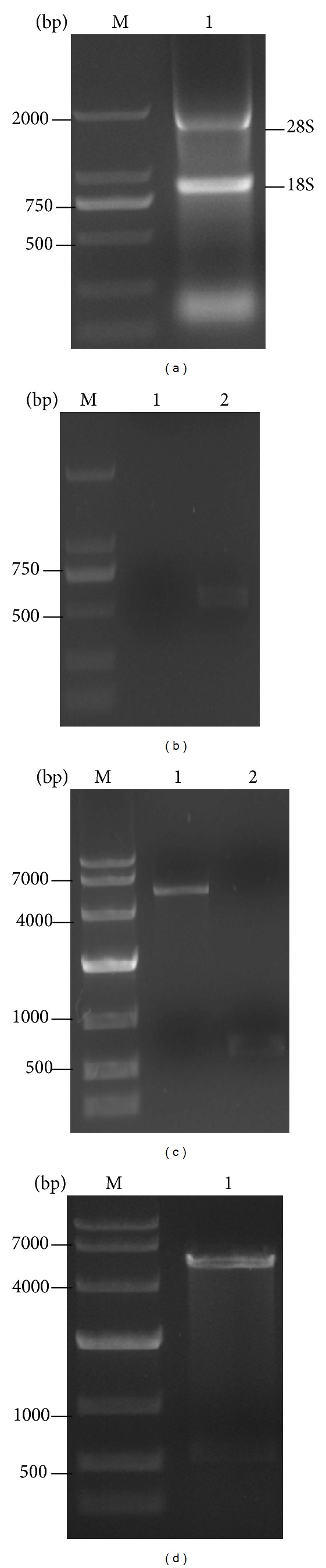
Construction and verification of the P-gp recombinant. (a) The total RNA of human colorectal cancer, M: DL2000 DNA marker; lane 1: total RNA of human colorectal cancer. (b) PCR amplification of P-gp_21_ gene, M: DL2000 DNA Marker; lane 1: blank control; lane 2: target gene of P-gp_21_. (c) pET28a (+) and P-gp_21_ gene digested by *EcoR I/Xho I* double enzyme, M: DL10000 DNA Marker; lane 1: the digested pET28a (+); lane 2: the digested P-gp_21_ target gene. (d) Positive clone of P-gp_21_ verified by double-enzyme cleavage, M: DL10000 DNA Marker; lane 1: positive clone of P-gp_21_.

To clone the MDR P-gp_21_, the P-gp transmembrane region (amino acid 784~968) adjoining the cytoplasmic nucleotide-binding domain (NBD) was chosen as the target according to its antigenicity predicted by using BepiPred 1.0b Server (Figure 3). After total RNA was extracted from human colorectal cancer, the target gene segments were then amplified successfully by PCR (Figures 4(a) and 4(b)). The target gene and pET28a (+) vector were digested by* EcoR I *and *Xho I* and collected by gel electrophoresis (Figure 4(c)). The target gene was cloned into pET28a (+) vector and transformed into BL21 (DE3) cell. The single clone was randomly picked and verified by* EcoR I *and *Xho I *digestion (Figure 4(d)). The sequence of the gene and its encoded amino acid of the positive clone were confirmed and compared using NCBI database. Although there are two nucleotides different from the retrieval sequence and no termination codon existed, its translated amino acid has no effect on its antigenicity (Table 1).

**Figure 5:**
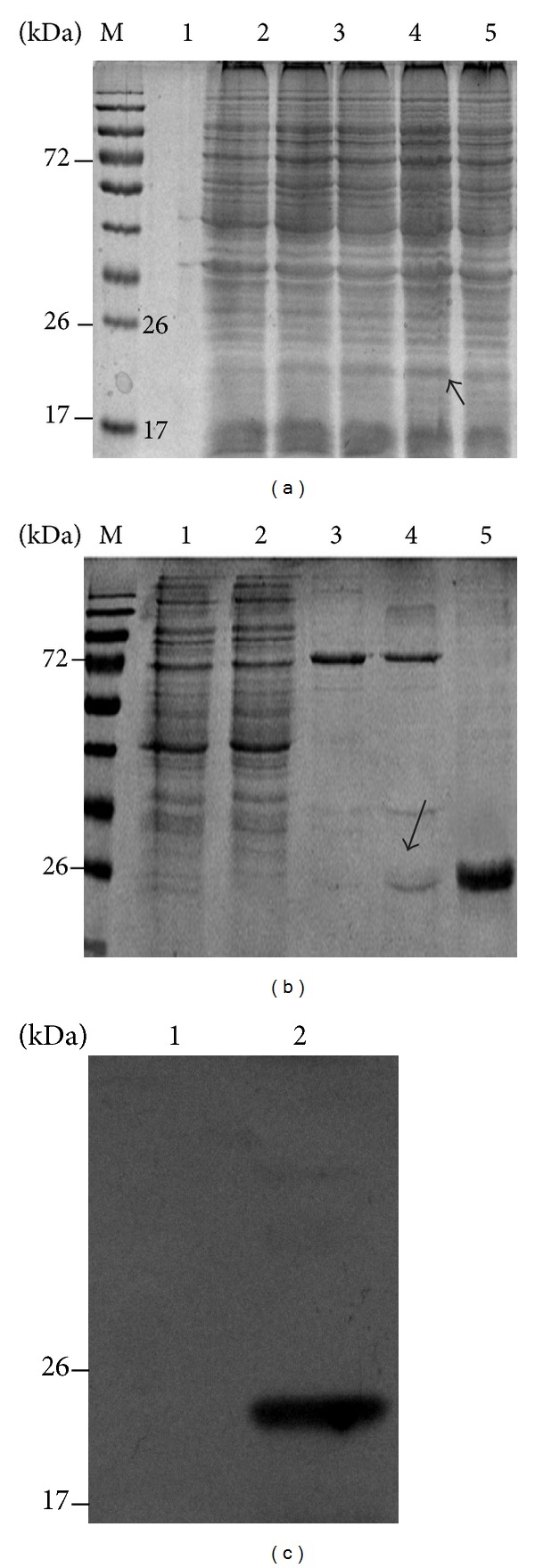
Expression, purification, and verification of the P-gp_21_. (a) SDS-PAGE image of the P-gp_21_ expression induced by IPTG: before induction (lane 1), induced for 1 hour (lane 2), induced for 2 hours (lane 3), induced for 3 hour (lane 4), and induced for 4 hours (lane 5); (b) purification of the P-gp_21_ protein by HiTrap HP, before passing Ni affinity column (lane 1), sample after passing Ni affinity column (lane 2), the washout of the Ni affinity column with 50 mM imidazole (lane 3), the washout of the Ni affinity column with 150 mM imidazole (lane 4), and the washout of the Ni affinity column with 300 mM imidazole (lane 5); (c) P-gp_21_ protein verified by Western blot: the recombinant with P-gp_21_ before induction (lane 1), purified P-gp_21_ protein (lane 2), antigen (P-gp_21_ protein), primary antibody (mouse anti-His antibody), and secondary antibody (HRP-labeled goat anti-mouse IgG antibody).

We optimized the production of P-gp_21_ and found that its expression was achieved maximally at 1.0 mM IPTG under 30°C for 6 hours (Figure 5(a)). The purified P-gp_21_ protein from the cultured bacteria was prepared at a final concentration of 5.2 *μ*g/*μ*L used for subsequent panning (Figure 5(b)). Meanwhile, the purified P-gp_21_ protein was verified by Western blot analysis as shown in Figure 5(c).

### 3.5. Biopanning and Identification of Fab Phage Antibody Library

After infection of the Fab antibody library by the helper phage VCSM13, the phage-displayed Fab antibody library was obtained, and the content was reached by 2.64 × 10^9^ pfu/*μ*L. The Fab phage antibody library was then screened by five rounds with the purified P-gp_21_ protein as shown in Table 2. The library was enriched by sequential panning using the P-gp_21_ immobilized on microplates. The number of colonies was calculated in CFU in each panning round, before and after selection by antigen, and the library enrichment factor was then estimated (Table 2). An enrichment factor of 34-fold was achieved in the second panning and only 0.12, 0.92, and 0.86 in the third, the forth, and the fifth panning, thus indicating that phage maximal production occurred in the second panning round. Ten phage clones were picked randomly after 5-round panning, and each plasmid DNA was extracted and digested by *Xba I/Sac I *and *Xho I/Spe I *(Figure 2(c)), respectively. The results show that compared with the primary Fab library, five-round panning made the insert ratio of the Fab chain increase from 72% to 100%.

### 3.6. Identification of the Optimal Clone Expressing Fab against P-gp_**21**_

**Figure 6:**
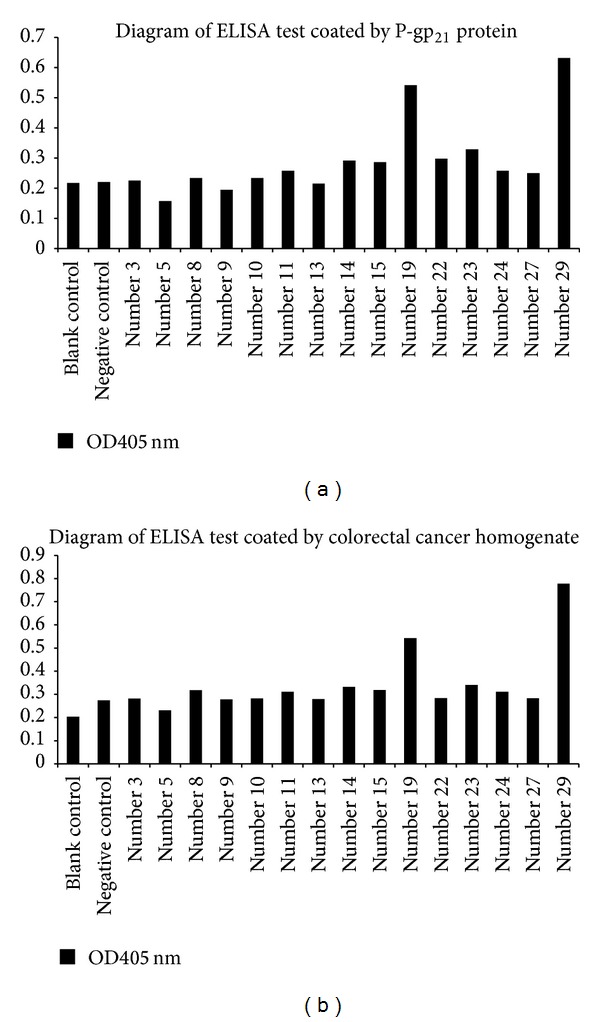
The optimal clone was checked by ELISA. (a) Diagram of ELISA (plate wells were coated by P-gp_21_ protein); (b) diagram of ELISA (plate wells were coated by colorectal cancer homogenate). The *x*-axis shows the clone number; the *y*-axis shows the ratio of the ELISA signal (A450) in the presence of 1 *μ*g/*μ*L purified P-gp_21_ and 2 *μ*g/*μ*L human colorectal cancer homogenate.

**Figure 7:**
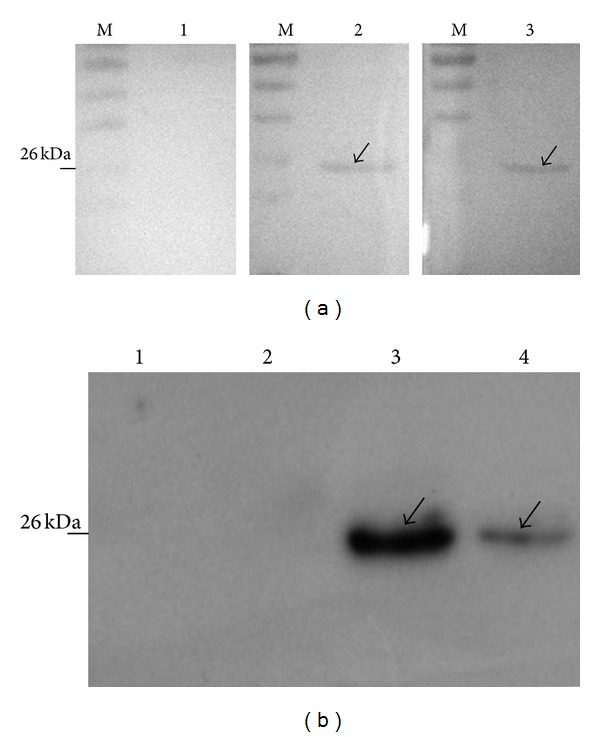
Western blot analysis of clone number 29. (a) 1: primary antibody: crude cell extract of pComb3 clone; 2: primary antibody: crude cell extract of number 29 clone; 3: primary antibody: mouse anti-His antibody; 1, 2, and 3: antigen: P-gp_21_ protein; secondary antibody: ALP-labeled horse anti-mouse IgG antibody. (b) 1: Antigen: BSA; 2: antigen: 15 kDa peptide; both 3 and 4: antigen: P-gp_21_ protein; 1, 2, 3, and 4: primary antibody: purified Fab; secondary antibody: HRP-labeled goat anti-mouse IgG antibody.

To identify the ideal clone expressing Fab against P-gp_21_, these positive clones were then amplified up to the same OD_600_; the expressed protein in the supernatant of each clone was further verified by ELISA. The expression level of the Fab in the supernatant from the cultured clones was examined when either the P-gp_21_ or the human colorectal cancer homogenate was used as an antigen. The results of the two ELISA tests showed identically that clone number 19 and number 29 were significantly higher than the control (Figure 6). However, only clone number 29 was further identified by Western blot analysis as an optimal clone when it was used as primary antibody (Figures 7(a) and 7(b)). Number 29 was sequenced for both light and heavy chains and aligned by a nucleotide blast program to confirm that the sequences with the higher identity were mouse IgG kappa chain and mouse immunoglobulin subtype of IgG2a, which are presented in Tables 3 and 4, respectively.

### 3.7. Production and Purification of the Soluble Fab Fragments

**Figure 8:**
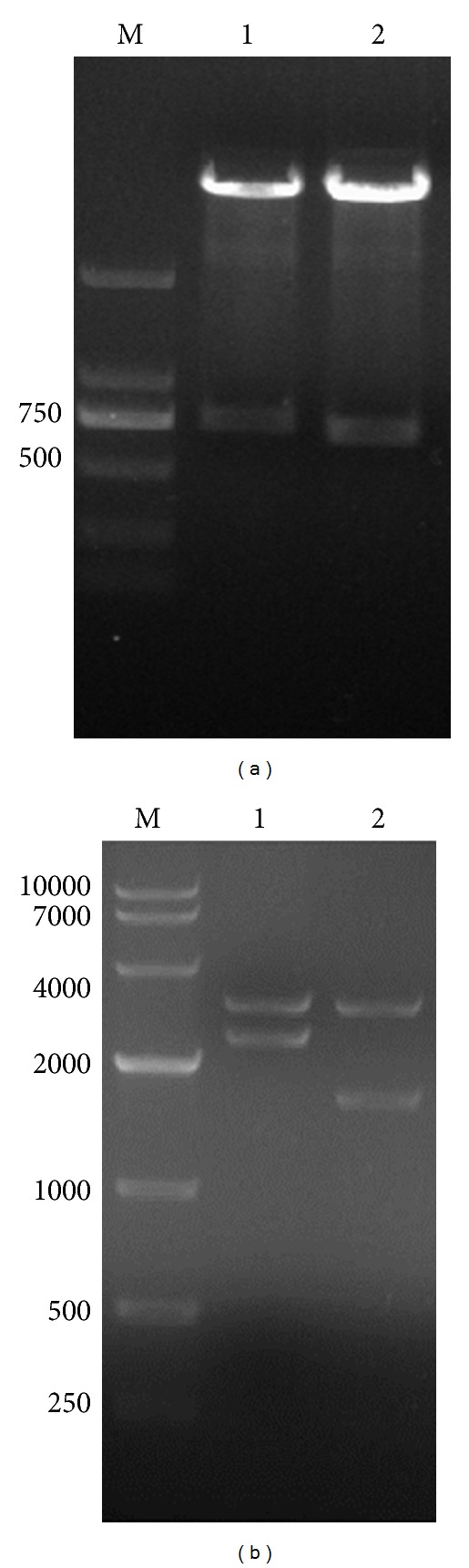
Verification of Fab soluble expression vector by *Not I* digestion. (a) M: marker DL2000; lanes 1-2: number 1 and number 2 clones were digested by *Spe I/Nhe I *for deleting gIII fragment. (b) M: marker DL10000; lane 1: recombinant with no gIII deletion digested by *Not I*; lane 2: recombinant after gIII deletion digested by *Not I*.

**Figure 9:**
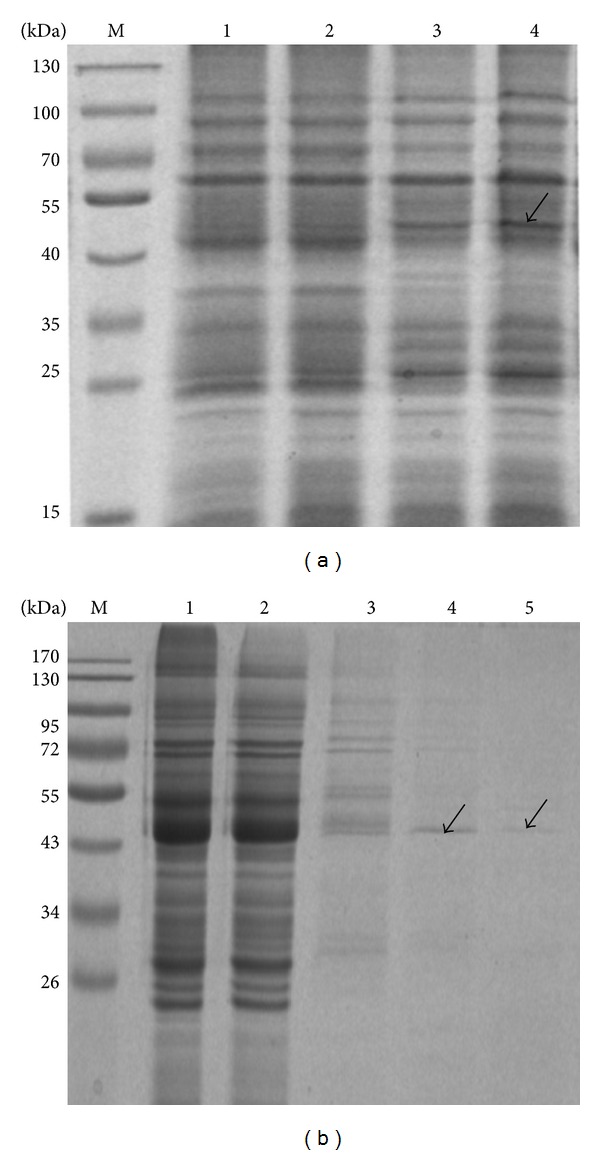
Fab expression and purification. (a) SDS-PAGE: 1, pComb3 clone was induced by 0.5 mM IPTG at 28°C for 8 h; 2, number 29 was not induced; 3, number 29 was induced by 0.5 mM IPTG at 28°C for 6 h; 4, number 29 was induced by 0.5 mM IPTG at 28°C for 8 h. (b) SDS-PAGE: 1, sample before passing HiTrap Protein L column; 2, sample after passing HiTrap Protein L column; 3, the washout of the HiTrap Protein L column with binding buffer; 4, the washout of the HiTrap Protein L column with sodium acetate buffer (pH 2.3).

The recombinant plasmid DNA samples of the clone number 29 were extracted and digested with *Spe I* and *Nhe I* to remove gIII (680 bp), which belongs to the bacteriophage (Figure 8(a)). The rescued fragment with 4700 bp was self-ligated and transformed into XL1-Blue cells. After the recombinant was identified by *Not I* digestion, the positive clone expressing Fab antibody was induced by IPTG (Figure 8(b)). Overexpression of the Fab antibody was achieved successfully under the optimized condition (Figure 9(a)). The Fab antibody from the crude cell extract was purified by affinity chromatography with recombinant protein L ligand owing to strong affinity for the variable region of an antibody's kappa light chain, showing that concentration of the Fab antibody at 150 mg/L was reached (Figure 9(b)). 

### 3.8. Characterization of the Anti-P-gp Fab Fragment Expressed in *E. coli* XL1-Blue

To further confirm the purified Fab, P-gp_21_ was detected and estimated to be 21 kDa in size when neither BSA nor 15-kDa peptide was used as the antigen by Western blot analysis (Figure 7(b)). 

**Figure 10:**
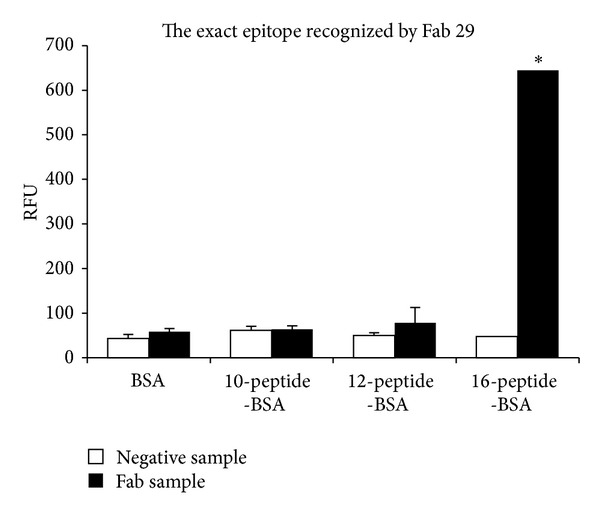
Identification of the exact epitope recognized by the anti-P-gp Fab by indirect immunofluorescence.

**Figure 11:**
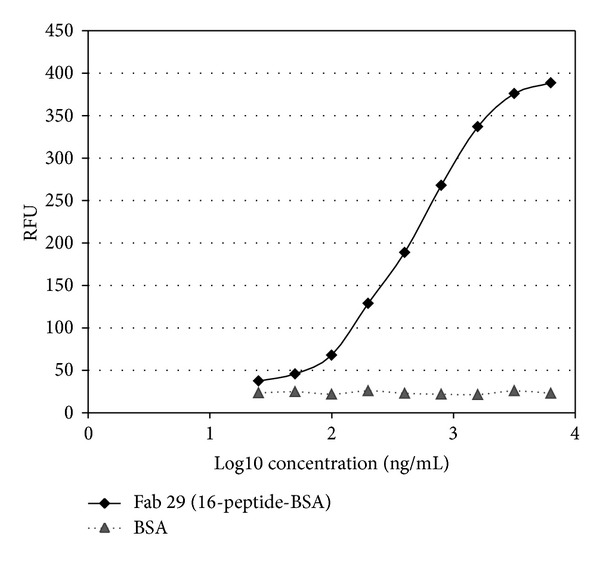
Binding activity assay of anti-P-gp Fab to 16-peptide-BSA and BSA by using indirect immunofluorescence.

Three peptides with strong antigenicity, which belong to P-gp_21_ coupled to bovine serum albumin, were synthesized and used for identifying the exact epitope recognized by the Fab using an indirect immunofluorescence assay. Compared to either BSA, 10-peptide-BSA, or 12-peptide-BSA, the Fab showed high specificity bound to 16-peptide-BSA (Figure 10). These results were further confirmed by indirect immunofluorescence assay as shown in Figure 11.

An aliquot of BSA, 10-peptide-BSA, 12-peptide-BSA, and 16-peptide-BSA, at 1 *μ*g/*μ*L, was coated on a 96-well microtiter plate. The anti-P-gp Fab (approximately 6 *μ*g/mL) was added and incubated at 37°C for 1 h. Fluorescein-isothiocyanate- (FITC-) conjugated goat anti-mouse IgG diluted 1 : 1000 is a secondary antibody for signal detection and crude cell extract of pComb3 cell was used as a negative control and secondary antibodies only as a background control. The relative fluorescence unit (RFU) was measured with an exitation spectrum at 490 nm and emission spectrum at 520 nm.

Data for anti-P-gp Fab and cell extract of pComb3 cell were subjected to ANOVA; differences between means were determined using the least significant difference (LSD) statistic (*P* < 0.05).

Microplates were coated with 16-peptide-BSA and BSA at 1 *μ*g/mL, respectively. Fab was titrated down by twofold dilution starting at 6 *μ*g/mL of antibody concentration. The second antibody was fluorescein-isothiocyanate- (FITC-) conjugated goat anti-mouse IgG. RFU was measured with an exitation spectrum at 490 nm and emission spectrum at 520 nm.

## 4. Discussion

Intrinsic or acquired resistance to a broad spectrum of chemotherapeutic agents often occurred in cancer patients who receive chemotherapy, resulting in failure of chemotherapy. The rate of tumor response to drug depends on a number of factors including pharmacokinetic factors, tumor type and biology, host response, drug sanctuaries, and drug resistance. Among them, multidrug resistance (MDR) may be the major obstacle affecting the effectiveness of the treatment. MDR occurrence varied among patients, of whom some are naturally resistant to multiple drug treatments (intrinsic resistance), while some may be induced by single drug treatment (acquired resistance) [15]. Due to cross-resistance, MDR limits the therapeutic efficacy of many important antitumor drugs. In this study, we engineered a novel antihuman colorectal cancer MDR gene (P-gp) Fab monoclonal antibody using the spleen tissue from the immunized mice. Because of the small size, without Fc regions, Fab antibody showed high specificity and sensitivity in detection of colorectal tumors with the MDR, suggesting that Fab antibody may be developed as a novel screening tool for personalized therapy of the patients who have colon cancer. 

Overexpression of P-gp is normally considered as the mechanism of the cytotoxic action of anticancer drugs [1, 16]. The P-gp protein has been developed as a biomarker used for detection of multiple drug resistance and monitoring chemotherapy in a number of studies [8, 17, 18].

A number of studies showed that complete IgG has some side effects due to FC fragments. The complete IgG has a long serum half-life resulting in poor contrast during the imaging process; additionally, inappropriate activation of the Fc receptor induces the cross-reactivity, giving rise to associated false positive signals, especially when it is used for FCM [9, 12, 19]. The size of the antibody molecules is an important parameter in analysis of pharmacokinetics and biodistribution. Compared to full-sized antibodies (with molecular weight 150 kDa), which have shown slow penetration of the solid tumors and nonuniform distribution, Fab fragments show rapid penetration to solid tumor and poor tumor retention. Meanwhile, due to the poor tissue penetration rate and the defeats of the cross-reactivity, the full-sized antibody is seldom used in medical applications [11, 12, 19, 20].

Intriguingly, although the difference in nucleotide sequence between the P-gp_21_ recombinant and the P-gp gene occurs in two sites, the antigenicity of three epitopes of P-gp_21_ was not affected. Thus, the Fab monoclonal antibody was generated successfully from clone number 29. 

A phage-displayed “library” is a heterogeneous mixture of such phage clones, each carrying a different foreign DNA insert and therefore displaying a different peptide on its surface. The phage antibody library technique is characterized by a large capacity and maximal diversity of antibody library, which is the primary guarantee for obtaining high bioactivity Fab fragments [13, 21]. 

The quantity, the diversity, and the mature degrees of the antibody library and the size of the library capacity can be affected by factors including (1) quality of RNA: high quality of total RNA (A260/A280 > 1.0) ensured the quality and the volume of the library; (2) maximal diversity: it can be done by tailoring more degenerate primers (17 Fd-primer pairs and 18 *κ*-primer pairs; 2 *λ*-primer pairs) from several donors and simultaneous construction of both the light chain library and the Fd library; and (3) cloning strategy: high efficiency of cloning and bacterial transformation are the key to achieve a Fab library attaining more than 10^6^ cfu of the recombinants.

In order to achieve a specific antibody against P-gp from the antibody library with low Fab recombinant frequency, the new panning strategy was used, which is to increase the washing time gradually during the five-round panning [11]. Phagemids may be enriched effectively during panning, and the specificity of the Fab displayed on the phagemid may be increased. Another important consideration for obtaining the high bioactivity phagemids is to use high purity of the P-gp_21_ antigen.

Overexpression of the P-gp protein also prevents stem cell from differentiation, leading to proliferation and amplification of the cell repertoire. The P-gp-caused MDR exists frequently in the residual tumor cells after chemotherapy and the tumor stem cells, inducing tumor metastasis [22, 23]. The drug-transporting property of stem cells conferred by the P-gp has been used as an important marker in isolation and analysis of cancer stem cells [24]. Understanding the mechanisms of cancer stem cell resistance to chemotherapy might therefore lead to discovery of a new therapeutic strategy for therapy of cancer [25, 26]. Therefore, detection of P-gp expression in tumor cells and suppression of the P-gp-mediated active efflux of chemotherapeutic drugs from the tumor cells may be used as an index for evaluation of chemotherapy [8, 27–29].

Improvement of the MDR sensitivity by targeting the P-gp has been extensively used as a strategy for therapy of cancer for more than 2 decades. Many agents modulating the function of the P-gp have been identified, including calcium channel blockers, calmodulin antagonists, steroidal agents, protein kinase C inhibitors, immunosuppressive drugs, antibiotics, and surfactants [25, 28, 30]. However, side effects of these agents often occur because of their low binding affinities. One of the critical points to be addressed is how to achieve clinically effective doses of these chemosensitizers in circulation without producing dose limited side effects. The use of the anti-P-gp MAbs as MDR-reversing agents may be the optimal approach to overcome MDR [31–33]. Moreover, targeting the P-gp by small-molecule compounds and/or antibodies is an effective strategy to overcome MDR in cancer [33, 34].

## 5. Conclusion

The peptide sized 21 kDa covering the P-gp transmembrane region was first prepared by us. And both anti-human colorectal cancer P-gp_21_ Fab antibody and its gene sequence have been successfully obtained by phage display technology. We found that the Fab antibody possessed better avidity either with human colorectal cancer or transmembrane domains of human P-gp_21_. After having been purified by HiTrap Protein L, the concentration of Fab antibody reaches 150 mg/L. The exact epitope of human P-gp which the Fab monoclonal antibody recognized by indirect immunofluorescence was ALKDKKELEGSGKIAT. Development of the specific Fab antibody against P-gp may provide an optimal strategy for effective administration of personalized therapy of colorectal cancer and an effective screening tool for intervention of multidrug resistance. 

## Figures and Tables

**Table 1:** BLAST of the P-gp_21_ sequence with that retrieved from NCBI.

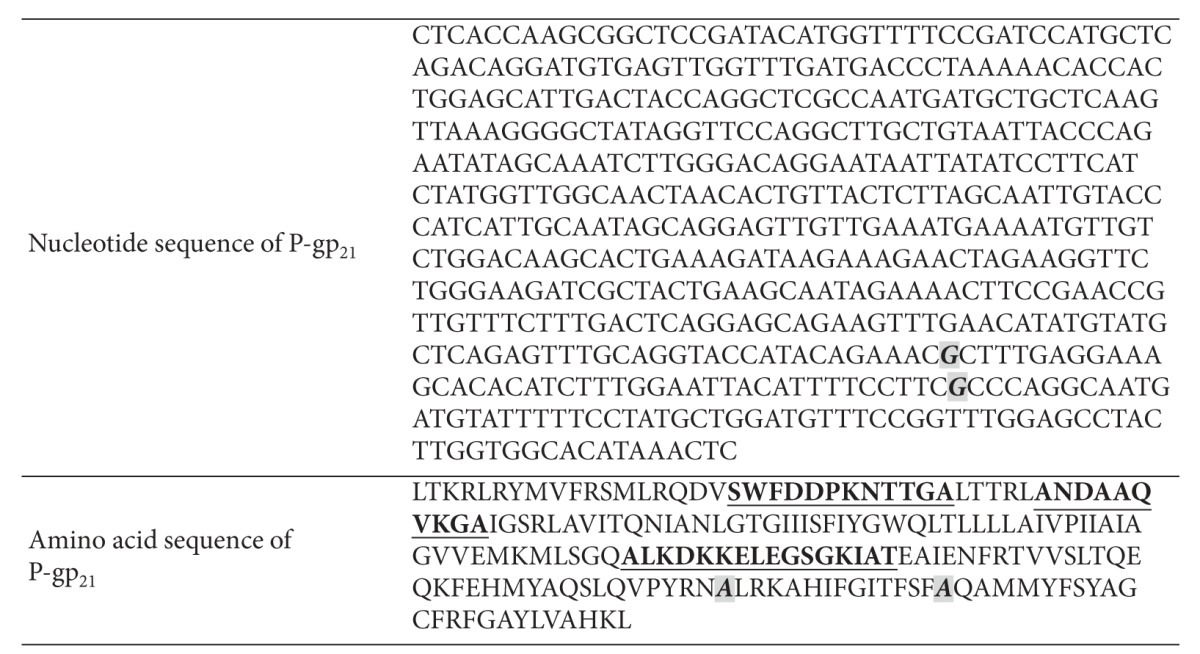

The sequences with underlines are three epitopes analyzed by BepiPred 1.0b Server; the nucleotides located as number 442 and number 484 (italics) were different from the retrieval sequences and their translated amino acid changed from serine to alanine as a result. The alanine located as number 148 and number 162 has no effect on the antigenicity of the three epitopes.

**Table 2:** The panning results of Fab phage antibody library.

Panning round	Phagemid input (CFU^a^/mL)	Phagemid output (CFU^b^/mL)	% bound (10^−2^)^c^	Enrichment^d^
1	1.3 × 10^13^	1.46 × 10^9^	1.10	
2	1.03 × 10^12^	3.90 × 10^9^	37.90	34.36
3	1.41 × 10^11^	6.38 × 10^7^	4.52	0.12
4	1.06 × 10^11^	4.40 × 10^7^	4.15	0.92
5	6.58 × 10^10^	7.70 × 10^6^	1.17	0.86

^a^Number of CFU (colony-forming units) of phagemids incubated with 16-peptide-BSA.

^b^Total number of CFUs of phage contained in elutes.

^c^(Phage output/phage input)/100.

^d^Fold increase in % bound compared to the previous round of panning.

**Table 3:** Light chain homology analysis of displaying number 29 clone the three sequences with higher homology (GenBank).

Nucleotide blast			

Query length: 635 Query ID: lcl∣10881 Mus musculus immunoglobulin kappa chain, constant region, mRNA^a^			

IgBLAST			
Identidade (total)	CDR1-IMGT	CDR2-IMGT	CDR3-IMGT

96.8% (274/283)	94.4% (17/18)	100% (9/9)	89.5% (17/19)

^a^GenBank: BC091754.1.

**Table 4:** Fd homology analysis of clone number 29 displaying the sequences with higher homology (GenBank).

Nucleotide blast			

Query length: 371 (CH1); query id: lcl∣31099 Mus musculus PAC-Fab mRNA for immunoglobulin gamma heavy chain variable region, partial cds^b^			
Query length: 696; query id: lcl∣62943 M. musculus mRNA for monoclonal antibody heavy chain gamma 2a^c^			

IgBLAST			
Identidade (total)	CDR1-IMGT	CDR2-IMGT	CDR3-IMGT

95.9% (282/294)	91.7% (22/24)	83.3% (20/24)	100% (6/6)

^b^GenBank: X70423.1.

^c^GenBank: AB678724.1.
