# Erratum to “Characterization of Human Colorectal Cancer MDR1/P-gp Fab Antibody”

**DOI:** 10.1155/2014/780429

**Published:** 2014-02-03

**Authors:** Xuemei Zhang, Gary Guishan Xiao, Ying Gao

**Affiliations:** ^1^Department of Biochemistry and Molecular Biology, Dalian Medical University, 9 Western Section, Lushun South Street, Lvshunkou District, Dalian 116044, China; ^2^The Medical College of Dalian University, Dalian Economic & Technical Development Zone, Dalian 116622, China; ^3^Departments of Medicine and Medical Microbiology, Creighton University, 601 N 30th Street, Omaha, NE 68131, USA

The affiliations of the authors in the original paper are corrected here.

